# Characterization of Novel Pathogenic Variants Leading to Caspase-8 Cleavage-Resistant RIPK1-Induced Autoinflammatory Syndrome

**DOI:** 10.1007/s10875-022-01298-2

**Published:** 2022-06-18

**Authors:** Alfonso José Tapiz i Reula, Alexis-Virgil Cochino, Andreia L. Martins, Diego Angosto-Bazarra, Iñaki Ortiz de Landazuri, Anna Mensa-Vilaró, Marta Cabral, Alberto Baroja-Mazo, María C. Baños, Zulema Lobato-Salinas, Virginia Fabregat, Susana Plaza, Jordi Yagüe, Ferran Casals, Baldomero Oliva, Antonio E. Figueiredo, Pablo Pelegrín, Juan I. Aróstegui

**Affiliations:** 1 Department of Internal Medicine, Fundació Althaia, Manresa, Spain; 2 University of Medicine and Pharmacy “Carol Davila,”, Bucharest, Romania; 3 National Institute for Mother and Child Health “Alessandrescu-Rusescu,”, Bucharest, Romania; 4 Department of Pediatrics, Hospital Prof. Doutor Fernando Fonseca, Amadora, Portugal; 5 Instituto Murciano de Investigación Biosanitaria IMIB-Arrixaca, Hospital Clínico Universitario Virgen de la Arrixaca, El Palmar, Murcia, Spain; 6 Department of Immunology, Hospital Clínic, Barcelona, Spain; 7 Institut d’Investigacions Biomèdiques August Pi i Sunyer, Barcelona, Spain; 8 Unit of Pediatric Rheumatology, Hospital Prof. Doutor Fernando Fonseca, Amadora, Portugal; 9 Department of Pediatrics, Fundació Althaia, Manresa, Spain; 10 School of Medicine, University of Barcelona, Barcelona, Spain; 11 Genomics Core Facility, Department of Experimental and Life Sciences, Universitat Pompeu Fabra, Parc de Recerca Biomèdica de Barcelona, Barcelona, Spain; 12 Departament of Genetics, Microbiology and Statistics, Faculty of Biology, University of Barcelona, Barcelona, Catalonia Spain; 13 Structural Bioinformatics Lab GRIB-IMIM, Department of Experimental and Life Sciences, Universitat Pompeu Fabra, Parc de Recerca Biomèdica de Barcelona, Barcelona, Spain; 14 Unit of Pediatric Immunology, Hospital Prof. Doutor Fernando Fonseca, Amadora, Portugal; 15 Department of Biochemistry and Molecular Biology B and Immunology, Faculty of Medicine, University of Murcia, Murcia, Spain

**Keywords:** Receptor-interacting kinases, RIPK1, necroptosis, autoinflammatory diseases, cleavage-resistant RIPK1-induced autoinflammatory syndrome

## Abstract

Pathogenic *RIPK1* variants have been described as the cause of two different inborn errors of immunity. Biallelic *loss-of-function* variants cause the recessively inherited RIPK1 deficiency, while monoallelic variants impairing the caspase-8-mediated RIPK1 cleavage provoke a novel autoinflammatory disease (AID) called cleavage-resistant RIPK1-induced autoinflammatory (CRIA) syndrome. The aim of this study was to characterize the pathogenicity of two novel RIPK1 variants located at the cleavage site of caspase-8 detected in patients with dominantly-inherited, early-onset undefined AID. *RIPK1* genotyping was performed by Sanger and next-generation sequencing. Clinical and analytical data were collected from medical charts, and in silico and in vitro assays were performed to evaluate the functional consequences. Genetic analyses identified two novel heterozygous *RIPK1* variants at the caspase-8 cleavage site (p.Leu321Arg and p.Asp324Gly), which displayed a perfect intrafamilial phenotype-genotype segregation following a dominant inheritance pattern. Structural analyses suggested that these variants disrupt the normal RIPK1 structure, probably making it less accessible to and/or less cleavable by caspase-8. *In vitro* experiments confirmed that the p.Leu321Arg and p.Asp324Gly RIPK1 variants were resistant to caspase-8-mediated cleavage and induced a constitutive activation of necroptotic pathway in a similar manner that previously characterized RIPK1 variants causing CRIA syndrome. All these results strongly supported the pathogenicity of the two novel RIPK1 variants and the diagnosis of CRIA syndrome in all enrolled patients. Moreover, the evidences here collected expand the phenotypic and genetic diversity of this recently described AID, and provide interesting data about effectiveness of treatments that may benefit future patients.

**Supplementary Information:**

The online version contains supplementary material available at 10.1007/s10875-022-01298-2.

## Introduction

Autoinflammatory diseases (AIDs) represent a special type of inborn errors of immunity (IEI) characterized by recurrent episodes of fever and sterile inflammation, which are a consequence of the overproduction of proinflammatory cytokines and increased cell death [1]. Different types of programmed cell death (PCD) have been molecularly characterized, with apoptosis, pyroptosis, and necroptosis as the most relevant ones [2, 3]. All types of PCD are physiological processes, essential for normal tissue development and homeostasis. However, an excessive cell death may amplify the inflammatory cascade and even provokes uncontrolled life-threatening events.

Necroptosis is a specific type of PCD triggered after the binding of certain cytokines (i.e., TNF) to cell surface receptors containing cytosolic death domains (DD). Unlike apoptosis and similarly to pyroptosis, necroptosis is associated with loss of membrane integrity, release of sterile intracellular content to the extracellular space, and the induction of a strong inflammatory response [4]. To prevent harmful reactions, necroptosis is tightly regulated, with the mixed-lineage kinase domain-like (MLKL), receptor-interacting serine/threonine kinase-1 (RIPK1), and RIPK3 proteins as its most important regulatory elements [2, 4]. Two different IEI have been associated with pathogenic variants in the *RIPK1* gene. On one side, the deficiency of RIPK1 is a recessively inherited disease characterized by severe immunodeficiency, arthritis, and early-onset inflammatory bowel disease that is a consequence of biallelic, *loss-of-function RIPK1* variants [5–8]. On the other side, dominantly-inherited variants at the amino acid residue Asp324 have been associated with the cleavage-resistant RIPK1-induced autoinflammatory (CRIA) syndrome, a recently described monogenic AID characterized by early-onset periodic fever and lymphadenopathies [9, 10].

In this study, we investigated three unrelated families suffering from a dominantly-inherited, early-onset undefined AID. Genetic analyses detected two novel heterozygous missense variants in the *RIPK1* gene, p.Leu321Arg and p.Asp324Gly, located at the consensus binding and cleavage site of caspase-8, which perfectly segregated with the disease in each family. In vitro studies revealed that both variants prevented the normal caspase-8-mediated RIPK1 cleavage in a similar manner to those variants already described as causing CRIA syndrome. Additional experiments strongly supported the pathogenicity of these novel RIPK1 variants. Altogether, these data establish the molecular and clinical diagnosis of CRIA syndrome in all enrolled patients.

## Methods

### Patients

Six patients from three unrelated families affected by a dominantly-inherited undefined AID were enrolled in this study. Patients’ data were registered at time of clinical diagnosis and/or at the time of genetic testing. Written informed consent was obtained from all adult individual participants included in the study (or from the parents/legal guardians of those participants under 16 years old). The Ethical Review Board of Hospital Clínic approved the study (Code HCB/2019/0631), which was performed in line with the principles of the 1964 Declaration of Helsinki and its later amendments.

### Molecular Genetics

Genomic DNA was extracted from whole blood using a QIAmp DNA Blood Mini Kit (QIAgen, Germany). Amplicons covering all exons and intronic boundaries of genes associated with monogenic AID (Supplementary Table S1) were generated by in-house designed polymerase chain reaction (PCR) amplification in an Access Array System 48.48 platform (Fluidigm, USA). Library preparation, control quality, and quantification were performed according to manufacturers’ instructions, and sequencing was performed on a NextSeq platform (Illumina, USA). Reads were mapped against the human reference genome build hg19 using the Burrows-Wheeler Aligner software [11], and bam and bai files were obtained. Reads analyses were performed with the SeqNext software (JSI Medical Systems, Germany).

All detected variants were confirmed with the Sanger method of DNA sequencing. Briefly, specific exons were PCR-amplified, purified with Illustra ExoStar 1-Step kit (GE Healthcare, USA), bidirectionally fluorescence sequenced using ABI BigDye® Terminator v3.1 Cycle Sequencing Kit (Applied Biosystems, USA) and run on an automated ABI 3730XL DNA analyzer (Applied Biosystems, USA). Sequences were analyzed using the SeqPilot software (JSI Medical Systems, Germany) and classified according to the consensus recommendations of the American College of Medical Genetics and Genomics (ACMG) and the Association for Molecular Pathology (AMP) [12].

### Sequence Alignment

The sequences of RIPK1 for different species were obtained from UniProtKB (https://www.uniprot.org/). The multiple alignments were performed using the CLUSTALW Omega software (https://www.ebi.ac.uk/Tools/msa/clustalo/).

### Plasmid Construction

The human RIPK1 (plasmid #78842) and RIPK3 (plasmid #78804) expression vectors were obtained from Addgene, USA. The different constructs of human RIPK1 variants were generated by overlapping PCR and cloned into pcDNA3.1/V5-His TOPO (Life Technologies, USA). Sequencing of all constructs was performed to confirm correct variant and the absence of unwanted variants. All the RIPK1 constructions contained the HA epitope at the N-terminal position.

### Cell Transfection and Stimulation

HEK293T and HeLa were cultured in DMEM/F12 and DMEM high glucose, respectively (Lonza, Switzerland) supplemented with 10% FCS (Life technologies, USA) [13]. Lipofectamine 2000 (Invitrogen, USA) was used to transfect the different plasmids following the manufacturer’s instructions. HeLa cells were transfected with the different human RIPK1 plasmids (wild type and mutant) together with human RIPK3. After 4 h of transfection, necroptosis was induced by 48-h incubation with 100 ng/mL of human recombinant TNF (Merck-Millipore, USA), 20 μM of z-IETD-FMK caspase-8 inhibitor (BD biosciences, USA), 500 nM of Inhibitor of Apoptosis Protein (IAP) antagonist BV6 (Merck-Millipore, USA), and a SMAC mimetic that blocks cIAP1 and XIAP interaction with initiator caspases and triggers their proteasomal degradation. Necroptosis was blocked by incubating with 1 μM of necrosulfonamide (NSA; Abcam, UK).

### In Vitro RIPK1 Cleavage

HEK293T cells were transfected with the human RIPK1 (wild type and mutant) plasmids. After 24 h, cells were lysed for 30 min on ice using cold lysis buffer (50 mM Tris-HCl pH 8.0, 150 mM NaCl, 2% Triton X-100, supplemented with 1:10 dilution of protease inhibitor mixture; Merck-Millipore, USA) and centrifuged at 16,000 g for 15 min at 4 °C. Total amount of protein present in the cell lysates was quantified using Bradford reagent (Merck-Millipore, USA). Forty microgram of total protein of each cell lysate containing human RIPK1 were incubated with 1 U of human recombinant active caspase-8 (Merck-Millipore, USA) for 45 min at 37 °C in a reaction solution containing 50 mM HEPES, pH 7.2, 50 mM of NaCl, 0.1% CHAPS, 10 mM EDTA, 5% glycerol, and 10 mM DTT. Cells lysates were then resolved in 4–12% precast Criterion polyacrylamide gels (Bio-Rad, USA) and transferred to nitrocellulose membranes (Bio-Rad, USA) by electroblotting. Membranes were probed with anti-HA rabbit monoclonal antibody from Cell Signaling Technologies, USA (1:2000) and horseradish peroxidase (HRP)-conjugated secondary antibody from GE Healthcare, USA (1:5000). Then, stripping was performed on the membrane and probed with HRP-anti-β-actin from Santa Cruz Biotechnology, USA (1:10000).

### Lactate Dehydrogenase (LDH) Release and Yo-Pro-1 Uptake

LDH release was measured in HeLa cells transfected with human RIPK1 (wild type and mutant) to determine necroptosis using the Cytotoxicity Detection Kit (Roche, Switzerland) following the manufacturer’s instructions. The results were expressed as the percentage of total LDH intracellularly present in control cells. Necroptosis was also followed by Yo-Pro-1 uptake, for that HeLa cells transfected with the different RIPK1 plasmids were incubated with 2.5 μM Yo-Pro-1 (Invitrogen, USA) for 5 min, and then fluorescence was determined at 485 ± 9/515 ± 9 nm (excitation/emission) in a Synergy Mx plate reader (BioTek, USA), and the percentage of Yo-Pro-1 was compared to cells treated with 0.1% triton X-100 for 5 min.

### Cytokine Profile

The serum concentrations of different cytokines were measured using a custom bead-based multiplex Luminex immunoassay (eBioscience, USA).

### Statistics and Reproducibility

Statistics were calculated with Prism software 9 (GraphPad Software, USA). For two-group and multiple comparisons, the Mann-Whitney test and the Kruskal-Wallis test with Dunn’s correction were used to determine the statistical significance, respectively. Data are shown as mean values and error bars represent standard error from the number of independent assays indicated in the figure legend. *p* values of less than 0.05 were considered statistically significant. *p* value is indicated as *p < 0.05; **p < 0.01; ***p < 0.001; ns, not significant (*p* > 0.05).

## Results

### Patients

**Fig. 1:**
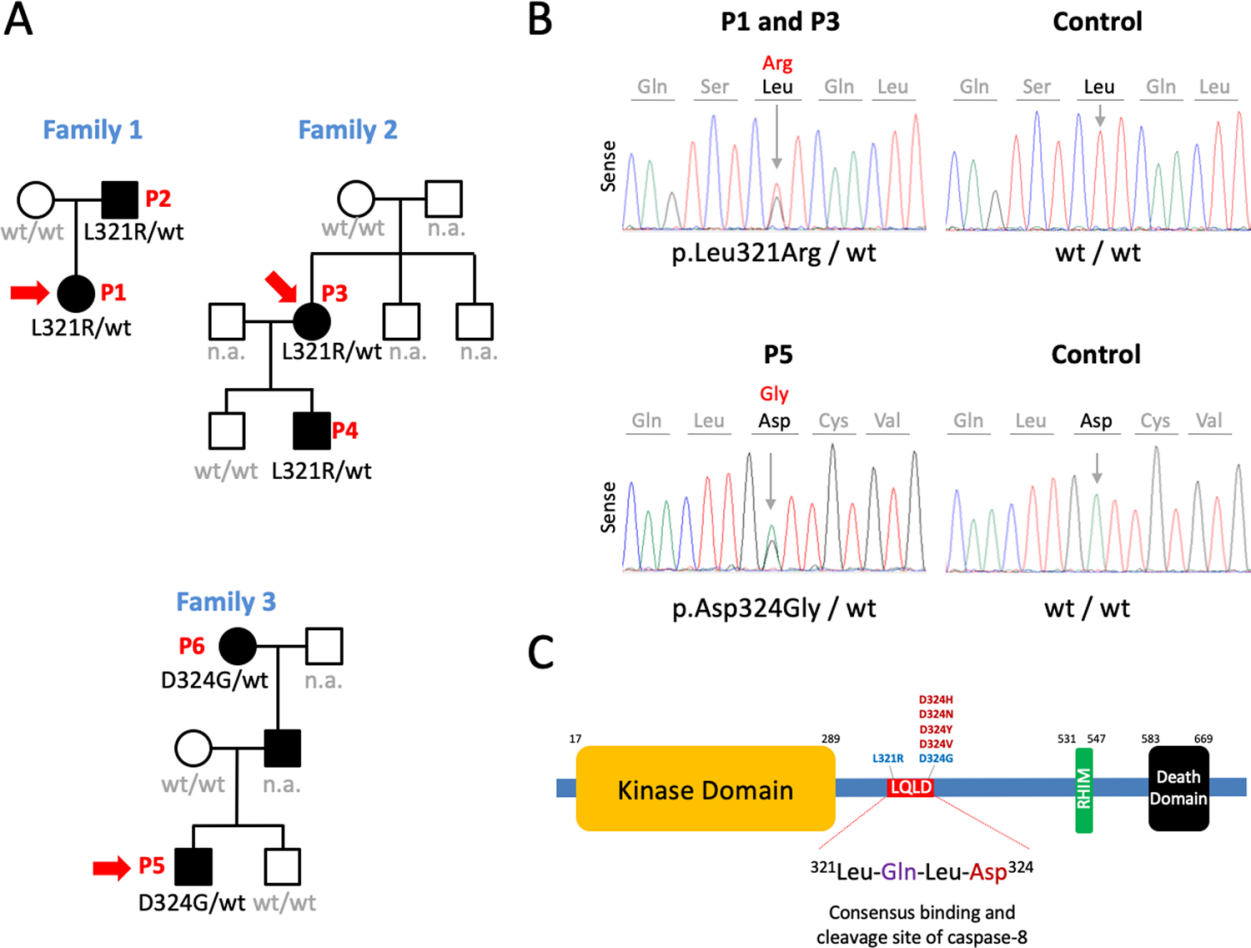
Novel variants in the *RIPK1* gene. **a** Pedigrees of enrolled families. Red arrows indicate the index patient of each family. Black symbols represent affected individuals, open symbols unaffected, squares males, and circles females. *RIPK1* genotypes are shown below each analyzed individual. wt, wild-type; n.a., not available. **b** Sense Sanger chromatograms from a healthy individual (control), and from patients P1–P3 and P5 carrying heterozygous genotypes for the p.Leu321Arg and p.Asp324Gly *RIPK1* variants, respectively. The gray arrows indicate the detected nucleotide exchanges. **c** Domains of human RIPK1. The sequence ^321^LQLD^324^ represents a consensus binding and cleavage site of human caspase-8. Reported pathogenic variants in residue Asp324 causing CRIA syndrome are displayed in red, and novel RIPK1 variants here described are shown in blue. RHIM, receptor-interacting protein (RIP) homotypic interaction motif

Among patients received in our department for genetic testing, we identified three unrelated families suffering from a similar inflammatory disease that was inherited as a dominant trait in each family (Fig. 1a). The disease started during the first months of life in most patients and displayed an episodic course, with episodes usually lasting less than 5 days and recurring every 0.5–1 month. The main clinical manifestations included fever (100%); lymphadenopathies (83%); chills (83%); arthralgias (83%); myalgias (83%); headache (83%); oral ulcers (67%), and pharyngitis (67%). Unlike other monogenic AID and IEI, a low incidence of recurrent infections, serositis, arthritis, and skin and ocular manifestations was observed. A detailed summary of patients’ manifestations and the outcome of administered treatments are shown in Table 1 and in the Supplementary Appendix. The results of routine laboratory tests collected during active disease (patients P3, P4, and P5) and during treatment with the anti-IL-1 drug canakinumab (patient P5) are shown in Supplementary Fig. S1.

**Table 1:** Clinical manifestation features of patients carrying novel *RIPK1* variants and comparison with reported patients with cleavage-resistant RIPK1-induced autoinflammatory syndrome (CRIA). Abbreviations: *CNS*, central nervous system; *MAS*, macrophage activation syndrome; *n.a.*, not administered; *n.r.*, not reported

		P1	P2	P3	P4	P5	P6	Reported CRIA patients
Gender		Female	Male	Female	Male	Male	Female	5 female/7 male
Current age		14 y	41 y	39 y	11 y	14 y	69 y	27.4 y (2–82 y)
Clinical data								
Age at disease onset		4 months	3 years	Birth	Birth	3 months	Childhood	1.8 months (0–6 months)
Febrile episodes	Temperature	38–40 °C	38–40 °C	39–40 °C	39–40 °C	39–40 °C	39–40 °C	40.5 °C (38.9–41 °C)
	Duration	2–5 days	2–5 days	2–5 days	2–5 days	2–7 days	2–3 days	< 3 days (n: 5) 3–5 days (n: 5) > 5 days (n: 1)
	Recurrence	1–2 weeks	1–2 weeks	3–4 weeks	3–4 weeks	1–2 weeks	3–4 weeks	2.6 weeks (1–4 weeks)
Lymphadenopathies		Yes	No	Yes	Yes	Yes	Yes	91.7%
Splenomegaly		No	No	No	No	Yes	No	58.3%
Hepatomegaly		No	No	No	No	Yes	Yes	25.0%
Abdominal Pain		No	No	No	Yes	No	No	41.7%
Nausea		Occasional	No	No	No	No	No	n.r.
Loss of appetite		Occasional	No	Yes	Yes	No	Yes	n.r.
Diarrhea		No	No	No	Occasional	No	No	n.r.
Oral Ulcers		Yes	Yes	Yes	Yes	No	No	71.4%
Pharyngitis		Occasional	Occasional	Occasional	No	No	Yes	42.9%
Genital Ulcers		No	No	No	No	No	No	0%
Pleuritis		No	No	No	No	No	No	n.r.
Pericarditis		No	No	No	No	Yes	No	n.r.
Chills		Yes (severe)	Yes (severe)	Yes	Yes	No	Yes	Some patients
Myalgias		Yes	Yes	Yes	Yes	No	Yes	n.r.
Arthralgias		Yes	Yes	Yes	Yes	No	Yes	37.5%
Arthritis		No	No	No	No	No	Yes	0%
Skin rash		No	No	No	Atopic dermatitis	No	No	0%
CNS manifestations		Headache	Headache, hallucinations	Headache	Headache	No	Headache	Some patients, not specified, with severe headaches or hallucinations
Papilledema		No	No	No	No	No	No	n.r.
Conjunctivitis		No	No	No	Occasional	No	No	n.r.
Uveitis		No	No	No	No	No	No	n.r.
AA amyloidosis		No	No	No	No	No	No	n.r.
MAS		No	No	No	No	Yes (MAS-like episode)	No	n.r.
Others		Exaggerated reactions to insect’s byte	Exaggerated reactions to insect’s byte	No	Allergy	No	No	n.r.
Response to treatments								
Colchicine		Negative	Negative	Partial	n.a.	Partial	Partial	Negative (6/6)
NSAIDs		Partial	Partial	Partial	Partial	Negative	Negative	n.r.
Corticoids		Positive	Positive	n.a.	n.a.	Partial	n.a.	Positive (7/7)
Anti-TNF		n.a.	Partial	n.a.	n.a.	n.a.	n.a.	Negative (3/4) Partial (1/4)
Anti-IL-1		n.a.	Negative	n.a.	n.a.	Partial (anakinra) Positive (canakinumab)	n.a.	Negative (4/4)
Anti-IL-6		n.a.	n.a.	n.a.	Positive	n.a.	n.a.	Partial (1/7) Positive (6/7)
Genetic data								
*RIPK1* genotype		p.Leu321Arg/wt	p.Leu321Arg/wt	p.Leu321Arg/wt	p.Leu321Arg/wt	p.Asp324Gly/wt	p.Asp324Gly/wt	p.Asp324His (n: 9) p.Asp324Asn (n: 1) p.Asp324Val (n: 1) p.Asp324Tyr (n: 1)

### Molecular Genetics

Analyses of genes associated with monogenic AID were performed in each family index patient (P1, P3, and P5) using a targeted gene panel (Supplementary Table S1), with all exons and intronic boundaries 100% covered with a minimum 50x coverage. These analyses gave negative results in all genes with the exception of the *RIPK1*, where two novel heterozygous missense variants, p.Leu321Arg (P1 and P3) and p.Asp324Gly (P5), were detected (Fig. 1b). Genetic evaluations performed in patients’ relatives only detected the heterozygous *RIPK1* variant genotype in affected individuals, thus confirming a perfect intrafamilial phenotype-genotype segregation (Fig. 1a). According to the ACMG/AMP recommendations, at the time of genetic testing, the p.Asp324Gly variant was classified as “likely pathogenic” and the p.Leu321Arg as “variant of uncertain significance” (Table 2).

**Table 2:** Features of novel *RIPK1* variants. ^1^Genome Build: GRCh37 / hg19; ^2^RefSeq: *RIPK1*: NM_003804.6. ^3^Classification of pathogenicity of gene variants performed on the consensus recommendations of the American College of Medical Genetics and Genomics (ACMG) and the Association for Molecular Pathology (AMP). Abbreviations: *Chr*, chromosome; *MAF*, minor allele frequency; *NHLBI-ESP*, National Heart, Lung and Blood Institute-Exome Sequencing Project; *gnomAD*, Genome Aggregation Database; *CSVS*, Collaborative Spanish Variant Server; *SIFT*, sorting intolerant from tolerant; *CADD*, Combined Annotation Dependent Depletion; *n.r.*, not registered; *VUS*, variant of uncertain significance; *LP*, likely pathogenic

Family	Families 1 and 2	Family 3
Structural features of variants		
Chromosome position^1^	Chr6: 3104505	Chr6: 3104514
Reference allele	T	A
Variant allele	G	G
Gene^2^	*RIPK1*	*RIPK1*
Exon	8	8
cDNA alteration	c.962T>G	c.971A>G
Predicted amino acid alteration	p.(Leu321Arg)	p.(Asp324Gly)
Population Genetics (MAF)		
1000 Genomes Project Phase 3 (2015 release)	0	0
NHLBI-ESP (ESP6500SI-V2 version)	0	0
Kaviar database (160204 version)	0	0
gnomAD (v2.1.1)	0	0
CSVS (3.0.1. version; February 2021 release)	0	0
Bioinformatics		
Polyphen-2 (Hum Var)	Probably damaging (1.00)	Probably damaging (0.917)
SIFT (Score)	Deleterious (0)	Deleterious (0.01)
MutationTaster	Disease causing	Disease causing
CADD PHRED	25.0	22.8
Phenotype-genotype databases		
ClinVar database	n.r.	n.r.
INFEVERS database	n.r.	n.r.
ACMG/AMP Variant Classification^3^	VUS	LP

CRIA syndrome has been associated with different RIPK1 variants located at amino acid residue Asp324 (p.Asp324His, p.Asp324Asn, p.Asp324Tyr, and p.Asp324Val) [9, 10]. The Asp324 residue is located in the sequence ^321^Leu-Gln-Leu-Asp^324^ (abbreviated ^321^LQLD^324^) of the RIPK1, which matches with the LXXD sequence that represents a consensus binding and cleavage site of caspase-8 (Fig. 1c) [14]. The two novel variants detected in this study are located at the ^321^LQLD^324^ sequence of the RIPK1 and are highly conserved across species (Supplementary Fig. S2). These evidences prompted us to hypothesize that these variants may have a similar behavior than those previously described as causing the CRIA syndrome, and additional investigations were performed to evaluate this hypothesis.

### Structural Modeling

Structural analyses of the human RIPK1 sequence located the residues Leu321 and Asp324 either in a coil or near α-helix regions, and in all analyzed models, these residues were surface-accessible in a flexible region out of a hydrophobic core (Supplementary Fig. S3a-c). RIPK1 sequence analysis by GraBCas software predicted either p.Leu321Arg or p.Asp324Gly amino acid substitutions completely abolished caspase-8 predicted cutting site at position 324 [15]. These results suggested that the p.Leu321Arg and p.Asp324Gly variants may lead to a potential defect of caspase-8-RIPK1 binding, which may potentially render the variant RIPK1 resistant to caspase-8 cleavage.

### Caspase-8-Induced Cleavage and Functional Analyses of RIPK1 Variants

**Fig. 2:**
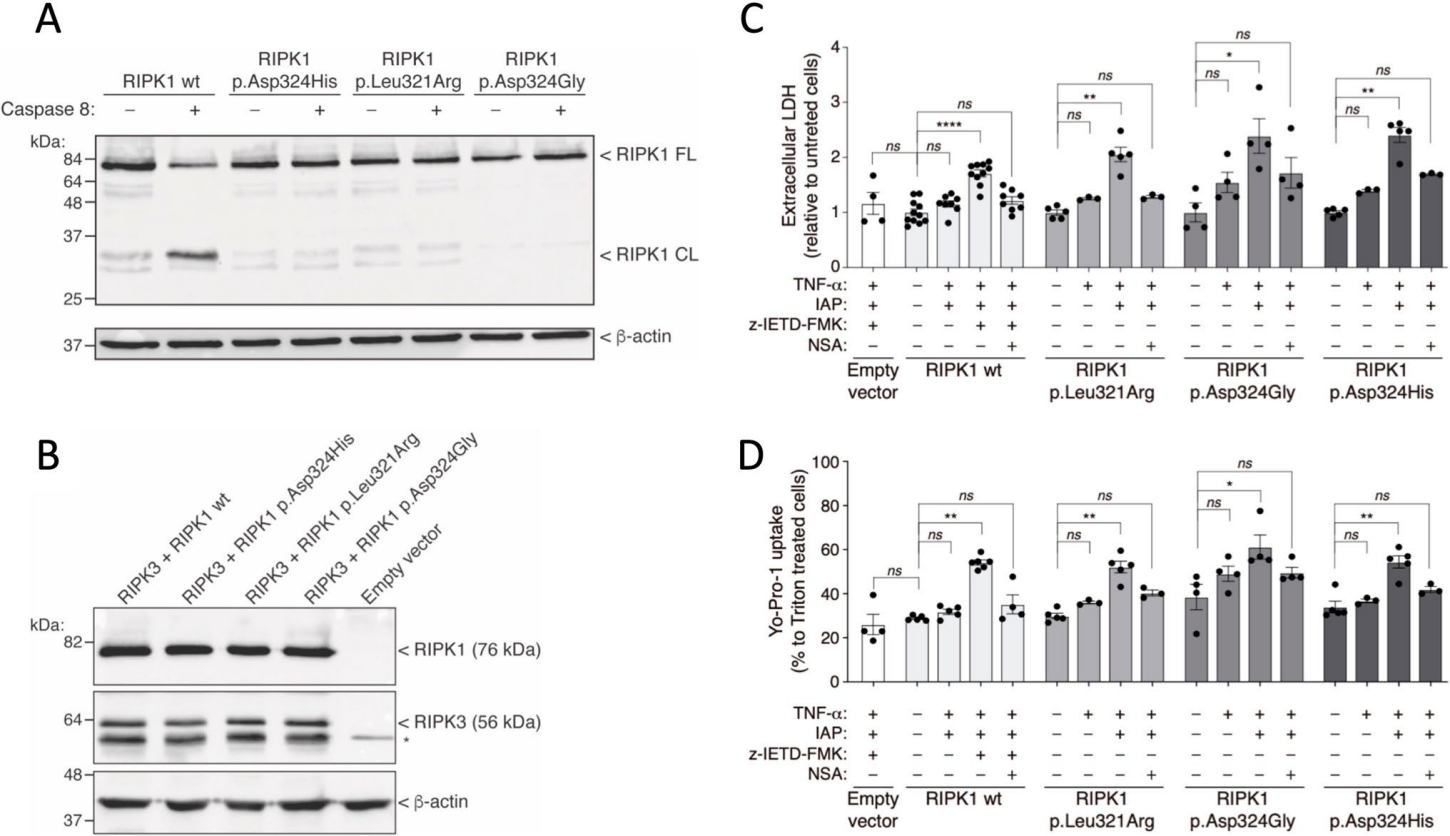
Functional analyses of novel RIPK1 variants. **a** Caspase-8 cleavage of RIPK1. Western blot for wild-type or variant RIPK1 expressed in HEK293T cells and incubated or not with recombinant caspase-8 as indicated. β-actin was used as a loading control. FL: full length; CL: cleaved. This Western blot is representative of three independent experiments. **b** Western blot showing the expression of RIPK3 and the different variants of RIPK1 in HeLa cells. β-actin was used as a loading control. Western blot is representative of three independent assays. Asterisks denote an unspecific band. **c**–**d** p.Leu321Arg and p.Asp324Gly RIPK1 variants induce cell death and cell permeabilization in response to TNF stimulation. Extracellular LDH release from HeLa cells (**c**) and Yo-Pro-1 uptake to HeLa cells (**d**) transfected with RIPK3 and empty pcDNA or the different RIPK1 variants after treatment with TNF and inhibitors of apoptosis proteins (IAP) antagonist, in the presence or absence of caspase-8 inhibitor (z-IETD-FMK) and/or necrosulfonamide (NSA); n: 4-8 different experiments. Each column represents the mean of all values, with bars in each column representing standard error; *denotes *p* < 0.05; **denotes *p* < 0.01; ***denotes *p* < 0.001; ****denotes *p* < 0.0001; Kruskal-Wallis test with Dunn’s multiple comparisons post-test

We analyzed whether the p.Leu321Arg and p.Asp324Gly variants impair the RIPK1 cleavage by caspase-8, which represents one of the most important regulatory mechanisms of its activity. In vitro experiments using HEK293T cells transfected with wild-type or variant RIPK1 revealed that both variants prevented RIPK1 processing by human recombinant caspase-8 in a similar manner to p.Asp324His RIPK1 variant, which has been previously characterized as a CRIA syndrome-causing variant (Fig. 2a) [9, 10].

We further assessed whether the novel RIPK1 variants were promoting necroptosis when expressed in HeLa cells (Fig. 2b). The expression of wild-type RIPK1 and RIPK3 in HeLa cells induced significant cell death and cell membrane permeabilization after treatment with TNF and inhibitors of apoptosis proteins (IAP) antagonist only when caspase-8 was blocked (Fig. 2c–d). These phenomena were blocked with NSA, a specific inhibitor of MLKL and necroptosis (Fig. 2c–d). When the RIPK1 variants (p.Leu321Arg and p.Asp324Gly) were employed in these assays, the cell death and membrane permeabilization after TNF and IAP antagonist treatment occurred at similar levels than those observed with the pathogenic p.Asp324His RIPK1 variant and were even higher than those observed in the wild-type RIPK1 construct in the presence of caspase-8 inhibitor (Fig. 2c–d). Moreover, NSA was also able to block cell death and membrane permeabilization induced by all three RIPK1 variants in the absence of caspase-8 inhibitor (Fig. 2c–d). These evidence suggested that the novel caspase-8 cleavage-resistant p.Leu321Arg and p.Asp324Gly RIPK1 variants were able to induce necroptosis.

### Profile of Circulating Cytokines

**Fig. 3:**
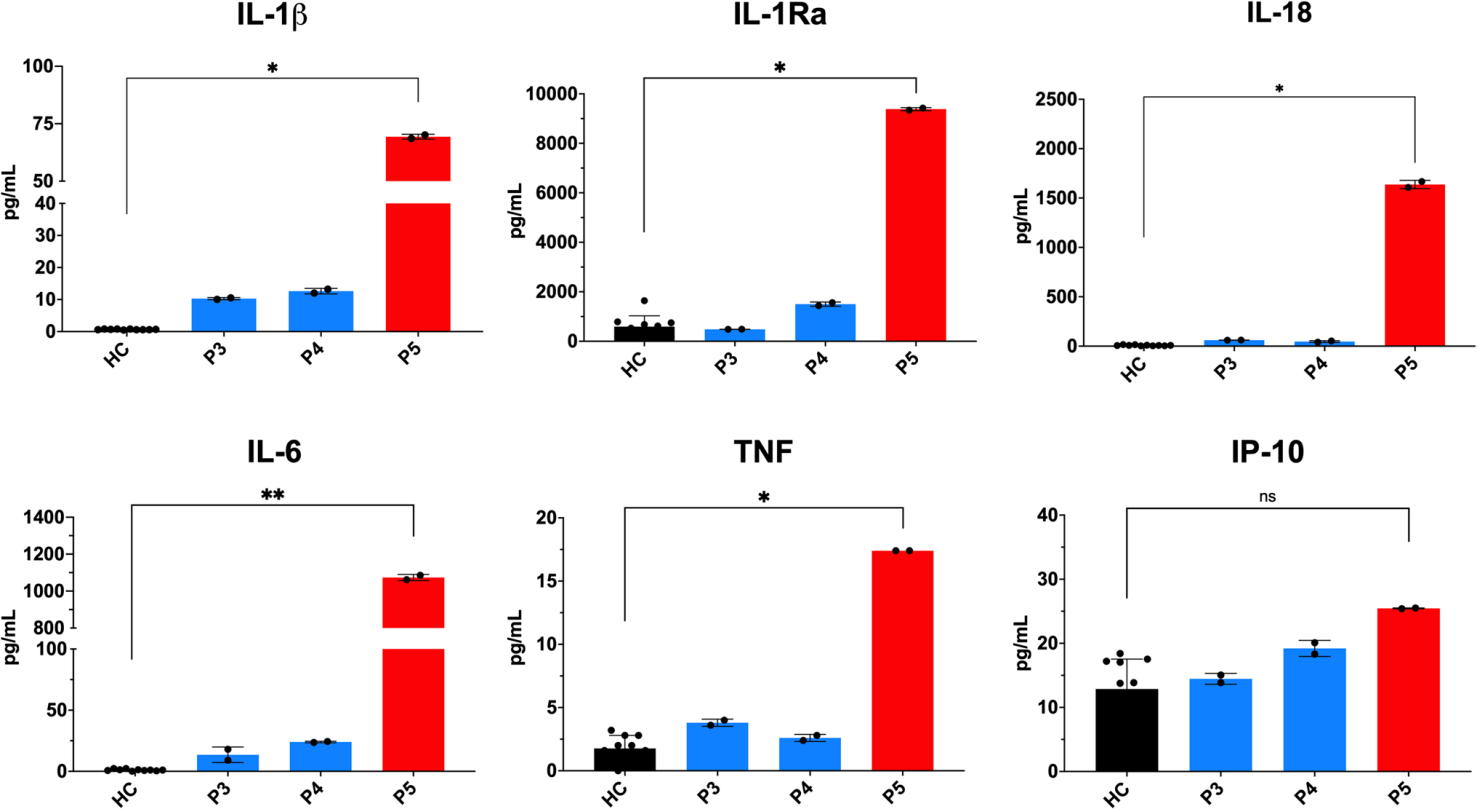
Serum levels of circulating cytokines in CRIA patients during inactive intervals (blue columns for patients P3 and P4) and during active disease (red column for patient P5) compared with healthy controls (HC; n: 10). Each dot represents the value of duplicate experiments of a collected serum. Each column represents the mean of all values, with bars in each column representing standard deviation (SD). n.s. denotes not significant; *denotes *p*<0.05; **denotes *p*<0.01

Finally, we investigated the profile of cytokines in different patients’ serum samples obtained during active disease (clinical manifestations and C-reactive protein >0.5 mg/dL) or during inactive intervals. We detected an increased production of different pro-inflammatory cytokines such as IL-6, IL-1β, and TNF in patient P5 during active disease when compared to healthy controls (HC). By contrast, the levels of these cytokines in those samples collected during inactive phases are markedly smaller than in active P5 and similar to HC (Fig. 3).

## Discussion

RIPK1 is a key regulator of different types of PCD, including necroptosis, and its activity can be down-regulated by the caspase-8. The caspase-8 binding and cleavage site occurs at the ^321^LQLD^324^ sequence of RIPK1, and the cleavage process separates the kinase domain from the intermediate and DD domains [14, 16]. A novel monogenic AID named CRIA syndrome has been described in twelve patients as a direct consequence of heterozygous variants at amino acid residue Asp324, located at the ^321^LQLD^324^ sequence [9, 10]. These variants render RIPK1 resistant to caspase-8-cleavage, provoke the loss of this regulatory mechanism, and enable its oligomerization, the RIPK3 recruitment, the MLKL phosphorylation, and the pore forming at cell membrane that finally led to both cell permeabilization and death. Therefore, a functional impairment in the caspase 8-mediated cleavage of RIPK1 resulting in exacerbated necroptosis represents the main mechanism of the pathogenesis of CRIA syndrome [9, 10].

In the present work, we have identified two novel missense variants in the ^321^LQLD^324^ sequence of the RIPK1 in patients with a dominantly inherited, undefined AID, which displayed a perfect intrafamilial phenotype-genotype relationship. On the basis of their location in the RIPK1, we hypothesized that these novel variants may be the cause of the observed manifestations and different experiments were performed to characterize their pathogenicity. Structural analyses revealed that the Leu321 and Asp324 residues were located in a surface-accessible region of the RIPK1, and the detected variants hypothetically provoke a conformational change and structural re-modeling of the protein, probably towards an α-helix conformation, making it less accessible to and/or less cleavable by caspase-8. Additional in vitro studies have clearly demonstrated that the two novel variants were caspase-8 cleavage-resistant, as those RIPK1 variants previously reported as causing the CRIA syndrome [9, 10]. Altogether, these evidences supported the pathogenicity of the novel RIPK1 variants here described and enabled us to establish the definitive diagnosis of CRIA syndrome in all enrolled patients increasing thus the total number of patients affected by this rare monogenic AID.

From a clinical point of view, the main manifestations detected in the enrolled patients are markedly similar to those previously described, with minor differences in the incidence of splenomegaly (16.7% vs 58.3); abdominal pain (16.7% vs 41.7%); oral ulcers (66.7% vs 71.4%); arthralgias (83.3% vs 37.5%), and headache (83.3% in our series vs not specified) [9, 10]. These data clearly indicate a relatively homogenous clinical picture for the CRIA syndrome and markedly different to the manifestations described in the deficiency of RIPK1, which is characterized by profound cellular immunodeficiency, with recurrent and severe infections, progressive polyarthritis, and early-onset inflammatory bowel disease [5–8].

The CRIA syndrome displays marked similarities with two well-known AIDs, the HIDS syndrome and the periodic fever, aphthosis, pharyngitis, and adenitis (PFAPA) syndrome, and consequently it should be considered in the differential diagnosis of these diseases. Some clinical features are frequent in all these three diseases, including early age at disease onset, the periodic nature of inflammatory episodes, and the presence of fever, lymphadenopathies, oral ulcers, and increased acute phase reactants [17, 18]. In contrast, there are some differences that may help clinicians to distinguish these entities. These differences include the inheritance pattern of the disease (dominant for the CRIA syndrome, recessive for HIDS, and polygenic inheritance for PFAPA); the near absence of inflammatory manifestations at serosa; skin and eye in CRIA syndrome; the tendency in PFAPA syndrome to spontaneously subside the inflammatory episodes during puberty and adolescence in contrast to HIDS and CRIA syndromes, where these episodes are still present during adulthood; certain differences in the hematological tests (tendency to normal values of leukocyte, neutrophil and platelet counts in CRIA syndrome); and the different outcome of frequently administered treatments. Finally, the deficiency of caspase-8 may be also considered in the differential diagnosis of CRIA syndrome, because both diseases share some pathophysiological mechanisms (partial or total impairment of normal caspase-8 function) and clinical features such as lymphadenopathies and splenomegaly [19–21]. However, the differences with regard to their inheritance pattern, age at disease onset, frequencies of severe infections and mucosal involvement, and mortality rates may help clinicians to clearly distinguish these two conditions. Table 3 includes a comparative summary of the main similarities and differences observed among the above mentioned diseases.

**Table 3:** Comparison of characteristics of RIPK1-associated diseases and similar autoinflammatory diseases. ^#^ 1/4 weeks denotes 1 episode every 4 weeks. Abbreviations: *RIPK1*, receptor-interacting serine/threonine kinase-1; *CRIA*, cleavage-resistant RIPK1-induced autoinflammatory syndrome; *HIDS*, hyper-IgD with periodic fever syndrome; *PFAPA*, periodic fever, aphthous stomatitis, pharyngitis and adenitis syndrome; *CNS*, central nervous system; *MAS*, macrophage activation syndrome; *APR*, acute phase reactants; *n.r.*, not reported

	RIPK1 deficiency [7–10] (n: 14)	CRIA [11, 12] (n: 12)	HIDS [19] (n: 114)	PFAPA [20] (n: 301)	Caspase 8 deficiency [21–23] (n: 5)
Inheritance	Recessive	Dominant	Recessive	Unknown	Recessive
Gene	*RIPK1*	*RIPK1*	*MVK*	None	*CASP8*
Disease onset < 5 years (%)	100%	100%	100%	90%	20%
Disease pattern	Continuous	Periodic	Recurrent (87%)	Periodic	Continuous
Fever (duration)	n.r.	3–5 days	4 days	4 days	Reported in 1 patient
Fever (recurrence)^#^	n.r.	1/1–4 weeks	1/4 weeks	1/4 weeks	Reported in 1 patient
Episodes triggering factors	n.r.	n.r.	45%	n.r.	n.r.
Recurrent infections	100% (Severe)	n.r.	n.r.	n.r.	100% (Severe)
IBD/abdominal pain/diarrhea	100% / n.r. / n.r.	0% / 41.7% / n.r.	Occasional / 88% / 84%	n.r. / 45% / 16%	n.r. / 40% / 40%
Oral ulcers	46%	71.4%	60%	57%	n.r.
Pharyngitis	n.r.	42.9%	28%	90%	n.r.
Arthralgias	n.r.	37.5%	71%	30%	n.r.
Arthritis	38%	n.r.	28%	3%	n.r.
Skin rash	21%	n.r.	54%	13%	40%
CNS manifestations	7%	Headache	Headache (38%)	4%	20%
Conjunctivitis	n.r.	n.r.	10%	5%	n.r.
Uveitis	n.r.	n.r.	2%	n.r.	n.r.
Lymphadenopathies	n.r.	91.7%	89%	78%	60%
Splenomegaly	31%	58.3%	n.r.	n.r.	80%
Asthma	n.r.	n.r.	n.r.	n.r.	40%
Chronic lung disease	7.1%	n.r.	n.r.	n.r.	60%
AA amyloidosis	n.r.	n.r.	4%	n.r.	n.r.
MAS	n.r.	n.r.	0.9%	n.r.	n.r.
Outcome	High mortality (46%)	Overall good (100%)	Occasional life-threatening complications	Overall good (100%)	High mortality (40%)

The precise mechanisms by which all these cytokines are increased in the CRIA syndrome are still under investigation. Previous evidences have shown that caspase-8 deficiency and defects of RIPK1 processing are connected with activation of NLRP3-inflammasome by different mechanisms [22–24], in particular by necroptosis-induced intracellular potassium efflux that is a well-established NLRP3 activator that triggers the activation of the inflammasome [22, 25]. Recent evidences also support the hypothesis of a direct connection between RIPK1 and the increase of inflammatory cytokines through the pyroptosis-related proteins gasdermin E (GSDME) and gasdermin D (GSDMD). Thus, it has been recently described that GSDME, a protein related with pyroptosis and IL-1β release, is activated in neutrophils in a RIPK1-dependent manner [26] and that the FADD-RIPK1-caspase 8 complex is recruited after infection to cleavage GSDMD and initiates the inflammatory cell death [27]. The loss of regulatory mechanisms of RIPK1 activity due to cleavage-resistant variants may generate an overproduction of inflammasome-related cytokines (IL-1β, IL-18), and inflammatory cell death through both necroptosis and pyroptosis, which in its turn may provoke the increase of plasma levels of IL-6 and TNF. This profile of circulating cytokines has similarities with those detected in other monogenic AIDs, specially associated with increased pyroptosis (inflammasomopathies, TNF-receptor-associated periodic syndrome), which are usually well controlled with anti-IL-1 and/or anti-TNF [28–30]. Unlike these diseases, patients with CRIA syndrome only displayed positive responses to the anti-IL-6 drug tocilizumab [9, 10]. To our knowledge, the patient P5 here described is the first one in whom the IL-1 blockade with the long-lasting monoclonal antibody canakinumab showed clear effectiveness to control the disease. The different effectiveness of treatments targeting inflammatory cytokines in CRIA syndrome will be a matter of future investigations that probably will require the identification of novel patients and the collection of additional data about inflammatory mediators. Our data on patients’ cytokine profiles has limitations due to the small sample size. Moreover, the serum samples were collected during active disease and inactive intervals and from patients with different RIPK1 mutant genotypes, which may lead to differences in the degree of necroptosis and the overproduction of specific cytokines. As CRIA is a rare disease and patients undergo various treatment modalities, defining the cytokine profile will be challenging. Nonetheless, our results are concordant with the limited data on circulating cytokines reported in CRIA syndrome [10].

In summary, we have shown herein the effectiveness of analyses based on next-generation sequencing methods to identify the genetic defects in patients with undiagnosed AID, as has been previously reported in other groups of AIDs and in other different diseases [31–35]. Our analyses identified two novel RIPK1 variants at the consensus sequence of caspase-8-mediated cleavage that lead to an exacerbated necroptosis. These evidences confirmed the diagnosis of CRIA syndrome in all enrolled patients and clearly differentiate them from patients with RIPK1 deficiency. Altogether, our results add novel data about the clinical and genetic diversity of the recently characterized CRIA syndrome. Before establishing the patients’ diagnosis, the treatments were empirically administered. With the novel data of patients’ cytokines profile during active disease, potential novel targeted treatments may be proposed for the treatment of future patients.

## Supplementary Information

ESM 1 (DOCX 24 kb)

ESM 2 (JPG 382 kb)

ESM 3 (JPG 1836 kb)

ESM 4 (JPG 1536 kb)

## Data Availability

Not applicable.
