# Correction to: Characterization of Novel Pathogenic Variants Leading to Caspase-8 Cleavage-Resistant RIPK1-Induced Autoinflammatory Syndrome

**DOI:** 10.1007/s10875-022-01322-5

**Published:** 2022-07-08

**Authors:** Alfonso José Tapiz i Reula, Alexis-Virgil Cochino, Andreia L. Martins, Diego Angosto-Bazarra, Iñaki Ortiz de Landazuri, Anna Mensa-Vilaro, Marta Cabral, Alberto Baroja-Mazo, María C. Baños, Zulema Lobato-Salinas, Virginia Fabregat, Susana Plaza, Jordi Yagüe, Ferran Casals, Baldomero Oliva, Antonio E. Figueiredo, Pablo Pelegrín, Juan I. Aróstegui

**Affiliations:** 1 Department of Internal Medicine, Fundacio Althaia, Manresa, Spain; 2 University of Medicine and Pharmacy “Carol Davila”, Bucharest, Romania; 3 National Institute for Mother and Child Health, “Alessandrescu-Rusescu”, Bucharest, Romania; 4 Department of Pediatrics, Hospital Prof. Doutor Fernando Fonseca, Amadora, Portugal; 5 Instituto Murciano de Investigacion Biosanitaria IMIB-Arrixaca, Hospital Clinico Universitario Virgen de la Arrixaca, El Palmar, Murcia, Spain; 6 Department of Immunology, Hospital Clinic, Barcelona, Spain; 7 Institut d’Investigacions Biomediques August Pi i Sunyer, Barcelona, Spain; 8 Unit of Pediatric Rheumatology, Hospital Prof. Doutor Fernando Fonseca, Amadora, Portugal; 9 Department of Pediatrics, Fundacio Althaia, Manresa, Spain; 10 School of Medicine, University of Barcelona, Barcelona, Spain; 11 Genomics Core Facility, Department of Experimental and Life Sciences, Universitat Pompeu Fabra, Parc de Recerca Biomedica de Barcelona, Barcelona, Spain; 12 Departament of Genetics, Microbiology and Statistics, Faculty of Biology, University of Barcelona, Barcelona, Catalonia Spain; 13 Structural Bioinformatics Lab GRIB-IMIM, Department of Experimental and Life Sciences, Universitat Pompeu Fabra, Parc de Recerca Biomedica de Barcelona, Barcelona, Spain; 14 Unit of Pediatric Immunology, Hospital Prof. Doutor Fernando Fonseca, Amadora, Portugal; 15 Department of Biochemistry and Molecular Biology B and Immunology, Faculty of Medicine, University of Murcia, Murcia, Spain

**Correction to: Journal of Clinical Immunology**

10.1007/s10875-022-01298-2

The article note is incorrect. It should have been stated as “Alfonso José Tapiz i Reula, Alexis-Virgil Cochino, Andreia L. Martins and Diego Angosto-Bazarra contributed equally to this work.”

The original version has been corrected.

