# Supplementary material for: Characterization of Novel Pathogenic Variants Leading to Caspase-8 Cleavage-Resistant RIPK1-Induced Autoinflammatory Syndrome
Source: J Clin Immunol. 2022 Jun 18;42(7):1421–32. doi: 10.1007/s10875-022-01298-2 (PMC9674708; doi:10.1007/s10875-022-01298-2)
Supplement: Supplementary file 1 — (DOCX 24 kb) [file 10875_2022_1298_MOESM1_ESM.docx]

**Characterization of Novel Pathogenic Variants Leading to Caspase-8 Cleavage-resistant RIPK1-induced Autoinflammatory Syndrome**

Supplementary Appendix

**Authors**

Alfonso José Tapiz i Reula MD, Alexis-Virgil Cochino MD, Andreia L. Martins MD, Diego Angosto-Bazarra PhD, Iñaki Ortiz de Landazuri BsC, Anna Mensa-Vilaró PhD, Marta Cabral MD, Alberto Baroja-Mazo PhD, María C. Baños BsC, Zulema Lobato-Salinas MD, Virginia Fabregat AS, Susana Plaza AS, Jordi Yagüe MD, PhD, Ferran Casals PhD, Baldomero Oliva PhD, Antonio E. Figueiredo MD, Pablo Pelegrín PhD, Juan I. Aróstegui MD, PhD

**Supplementary Information of Treatments Administered to patient P5**

Patient P5 is a 14-year-old boy who was born to non-consanguineous parents of Portuguese ancestry. His disease started at the age of 3 months, and genetic studies for inborn errors of immunity performed at the time, including monogenic autoinflammatory diseases, were negative. His definitive diagnosis of CRIA syndrome was established in 2021 once genetic and functional studies revealed a novel RIPK1 variant leading to an exacerbated necroptosis as a consequence of a caspase-8-mediated non-cleavable RIPK1 protein. Prior to this diagnosis the patient had received different treatment. He was treated with daily oral colchicine (1-2 mg q1d), non-steroidal anti-inflammatory drugs, and corticoids that resulted in null of mild improvement of some features, mainly the fever, but no normalization of laboratory parameters. On the basis of patient’s disease severity, and after obtaining the patient’s parents' approval, the referral pediatric rheumatologists started treatment with the recombinant IL-1 receptor antagonist (anakinra; dose 2.5mg/kg/day; subcutaneously). However, no clinical improvement was achieved with this treatment, and laboratory parameters still indicated weak control of subclinical inflammation: Haemoglobin: 10.5 g/dL; Erythrocyte Sedimentation Rate: 37-55 mm/h; C-reactive protein: 5.3-8.6 mg/dL.

Then, the clinical team decided to start treatment with the long-lasting anti-IL-1 monoclonal antibody canakinumab in monotherapy on June 26^th^, 2016 (2 mg/kg q8w; subcutaneously). On March 27^th^, 2017, the dosage was raised to 4 mg/kg q8w. During canakinumab treatment the acute inflammatory episodes became dramatically less frequent than at the initial dose, and even less frequent after increasing the canakinumab dose. Nearly all lab parameters normalized during canakinumab treatment.

Interestingly, during the treatment with canakinumab the patient experienced two episodes of infectious illnesses (a bacterial amygdalopharyngitis infection, and a SARS-Cov-2 infection), with a good recovery at home on both occasions. Finally, on June 21^st^, 2019, the patient was hospitalized due to a macrophage activation syndrome-like episode of unknown origin, which was successfully resolved with 5 pulses of methylprednisolone (30 mg/kg).

**Methods**

**Structural Analyses**

The amino acid sequence of the human RIPK1 protein was selected in the UniProtKB database (code Q13546). The servers iTASSER and Robetta were employed to obtain all potential structural models of the full RIPK1 sequence [1-2]. In addition, the online version of AlphaFold2 was employed to model the C-terminal region of RIPK1 (amino acid residues 301-670) [3].

**References**

1. Zheng W, Zhang C, Li Y, Pearce R, Bell EW, Zhang Y. Folding non-homologous proteins by coupling deep-learning contact maps with I-TASSER assembly simulations. Cell Rep Methods 2021; 1: 100014.
2. Yang J, Anishchenko I, Park H, Peng Z, Ovchinnikov S, Baker D. Improved protein structure prediction using predicted interresidue orientations. Proc Natl Acad Sci U S A 2020; 117: 1496-1503.
3. Online version of AlphaFold2 at: https://www.getmoonbear.com/AlphaFold2Lite.

**Legends of Supplementary Figures**

**Supplementary Figure S1. Results of Hematological and Biochemical Parameters in Patients’ Routine Laboratory Tests.** In P5, values obtained during active disease (P5act) or during treatment with canakinumab (P5canak) are depicted. Each dot represents the value obtained at an individual blood collection, with the horizontal line indicating the mean of all values. The normal range for each parameter is indicated by the horizontal blue square. *denotes p<0.05; **denotes p<0.01; ns, not significant.

**Supplementary Figure S2.** **Protein Sequence Alignment of RIPK1 in Different Animal Species**. Alignment showing the conservation of the key residues of the caspase-8 cleavage site present in RIPK1: Leu321 (L, red arrow) and Asp324 (D, blue arrow).

**Supplementary Figure S3. Structural Analyses of RIPK1 Variants at the ^321^LQLD^324^ Sequence.** RIPK1 models were obtained with iTasser (Panel a), Robetta (Panel b), and AlphaFold2 (Panel c). The dimer structure through the N-terminal region of RIPK1 is shown in gray and the model of the C-terminal region is shown in blue (iTasser), green (Robetta), and magenta (AlphaFold2). The region of amino acids 321-324 is shown in yellow.

**Supplementary Table S1. List of analyzed genes associated with autoinflammatory diseases.** Abbreviations: ADA2, adenosin deaminase 2; ARPC1B, actin related protein C1B; NOCARH, Neonatal-onset cytopenia, autoinflammation, rash and hemophagocytic lymphohistiocytosis; IL-10, interleukin-10; DIRA, deficiency of interleukin-1 receptor antagonist; DITRA, deficiency of interleukin-36 receptor antagonist; FMF, familial Mediterranean Fever; PAAND, Pyrin-associated autoinflammation with neutrophilic dermatosis; MK, mevalonate kinase; AIFEC, Autoinflammation with infantile enterocolitis; NAIAD, NLRP1-associated autoinflammation with arthritis and dyskeratosis; FCAS2, familial cold-induced autoinflammatory syndrome type 2; CAPS, cryopyrin-associated periodic syndromes; PLAID, PLCG2-associated antibody deficiency, and immune dysregulation; APLAID, Autoinflammation and PLCG2-associated antibody deficiency, and immune dysregulation; CANDLE, Chronic atypical neutrophilic dermatosis with lipodystrophy and elevated temperature; PRAAS, Proteasome-associated autoinflammatory syndrome; PAPA, Pyogenic arthritis, pyoderma gangrenosum and acne; Hz/Hc, Hyperzincemia and hypercalprotectinemia syndrome; HOIL-1, Heme-oxidized IRP2 ubiquitin ligase 1; CRIA, cleavage-resistant RIPK1-induced autoinflammatory syndrome; HOIP, HOIL-1-interacting protein; SAVI, STING-associated vasculopathy with onset in infancy; TRAPS11, TNFRSF11A-associated periodic syndrome; TRAPS, TNF Receptor I-associated periodic syndrome; SIFD, Sideroblastic anemia, B-cell immunodeficiency, periodic fevers, and developmental delay; PFIT, periodic fever, immunodeficiency, and thrombocytopenia syndrome.

| **Gene** | **Disease** | **Ref Seq** |  | **Gene** | **Disease** | **Ref Seq** |
| --- | --- | --- | --- | --- | --- | --- |
| *ADA2* | ADA2 Deficiency | NM_001282225.1 |  | *PLCG2* | PLAID-APLAID | NM_002661.3 |
| *ADAR* | Aicardi-Goutières syndrome Type 6 | NM_001111.5 |  | *POMP* | CANDLE/PRAAS | NM_015932.5 |
| *AP1S3* | Pustular Psoriasis | NM_001039569.1 |  | *PSMA3* | CANDLE/PRAAS | NM_002788.3 |
| *ARPC1B* | ARPC1B Deficiency | NM_005720.4 |  | *PSMB4* | CANDLE/PRAAS | NM_002796.2 |
| *CARD14* | Pustular Psoriasis | NM_024110.4 |  | *PSMB8* | CANDLE/PRAAS | NM_148919.3 |
| *CDC42* | NOCARH | NM_001791.4 |  | *PSMB9* | CANDLE/PRAAS | NM_002800.4 |
| *IFIH1* | Aicardi-Goutières syndrome Type 7 | NM_022168.3 |  | *PSMG2* | CANDLE/PRAAS | NM_020232.4 |
| *IL10* | IL-10 Deficiency | NM_000572.2 |  | *PSTPIP1* | PAPA-Hz/Hc | NM_003978.3 |
| *IL10RA* | IL-10R1 Deficiency | NM_001558.3 |  | *RBCK1* | HOIL-1 Deficiency | NM_031229.3 |
| *IL10RB* | IL-10R2 Deficiency | NM_000628.4 |  | *RELA* | Rel-A Haploinsufficiency | NM_021975.3 |
| *IL1RN* | DIRA | NM_173842.2 |  | *RIPK1* | CRIA | NM_003804.5 |
| *IL36RN* | DITRA | NM_173170.1 |  | *RNASEH2A* | Aicardi-Goutières syndrome Type 4 | NM_006397.2 |
| *LACC1* | Lacasse Deficiency | NM_153218.3 |  | *RNASEH2B* | Aicardi-Goutières syndrome Type 2 | NM_024570.3 |
| *LPIN2* | Majeed syndrome | NM_014646.2 |  | *RNASEH2C* | Aicardi-Goutières syndrome Type 3 | NM_032193.3 |
| *MEFV* | FMF-PAAND | NM_000243.2 |  | *RNF31* | HOIP Deficiency | NM_017999.4 |
| *MVK* | MK Deficiencies | NM_000431.3 |  | *SAMHD1* | Aicardi-Goutières syndrome Type 5 | NM_015474.3 |
| *NCSTN* | Hidradenitis suppurativa | NM_015331.2 |  | *TMEM173* | SAVI | NM_198282.3 |
| *NLRC4* | AIFEC | NM_021209.4 |  | *TNFAIP3* | A20 Haploinsufficiency | NM_006290.3 |
| *NLRP1* | NAIAD | NM_033004.3 |  | *TNFRSF11A* | TRAPS11 | NM_003839.3 |
| *NLRP12* | FCAS2 | NM_144687.2 |  | *TNFRSF1A* | TRAPS | NM_001065.3 |
| *NLRP3* | CAPS | NM_001243133.1 |  | *TREX1* | Aicardi-Goutières syndrome Type 1 | NM_033629.5 |
| *NOD2* | Blau syndrome | NM_022162.2 |  | *TRNT1* | SIFD | NM_182916.2 |
| *OTULIN* | Otulipenia | NM_138348.5 |  | *WDR1* | PFIT | NM_017491.4 |
