# Supplementary figures and images for: Characterization of Novel Pathogenic Variants Leading to Caspase-8 Cleavage-Resistant RIPK1-Induced Autoinflammatory Syndrome
Source: J Clin Immunol. 2022 Jun 18;42(7):1421–32. doi: 10.1007/s10875-022-01298-2 (PMC9674708; doi:10.1007/s10875-022-01298-2)

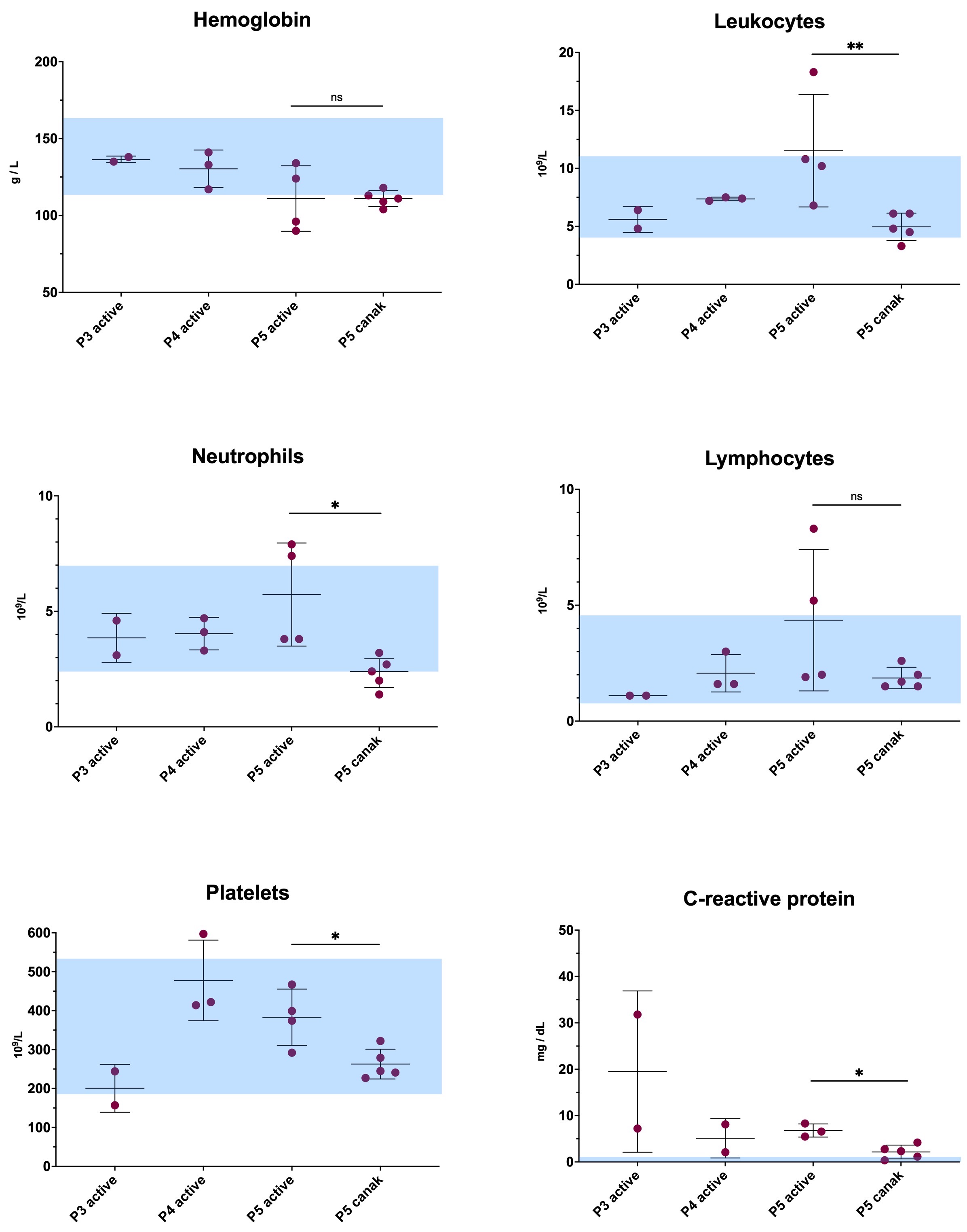

Supplement: Supplementary file 2 — (JPG 382 kb) [file 10875_2022_1298_MOESM2_ESM.jpg]

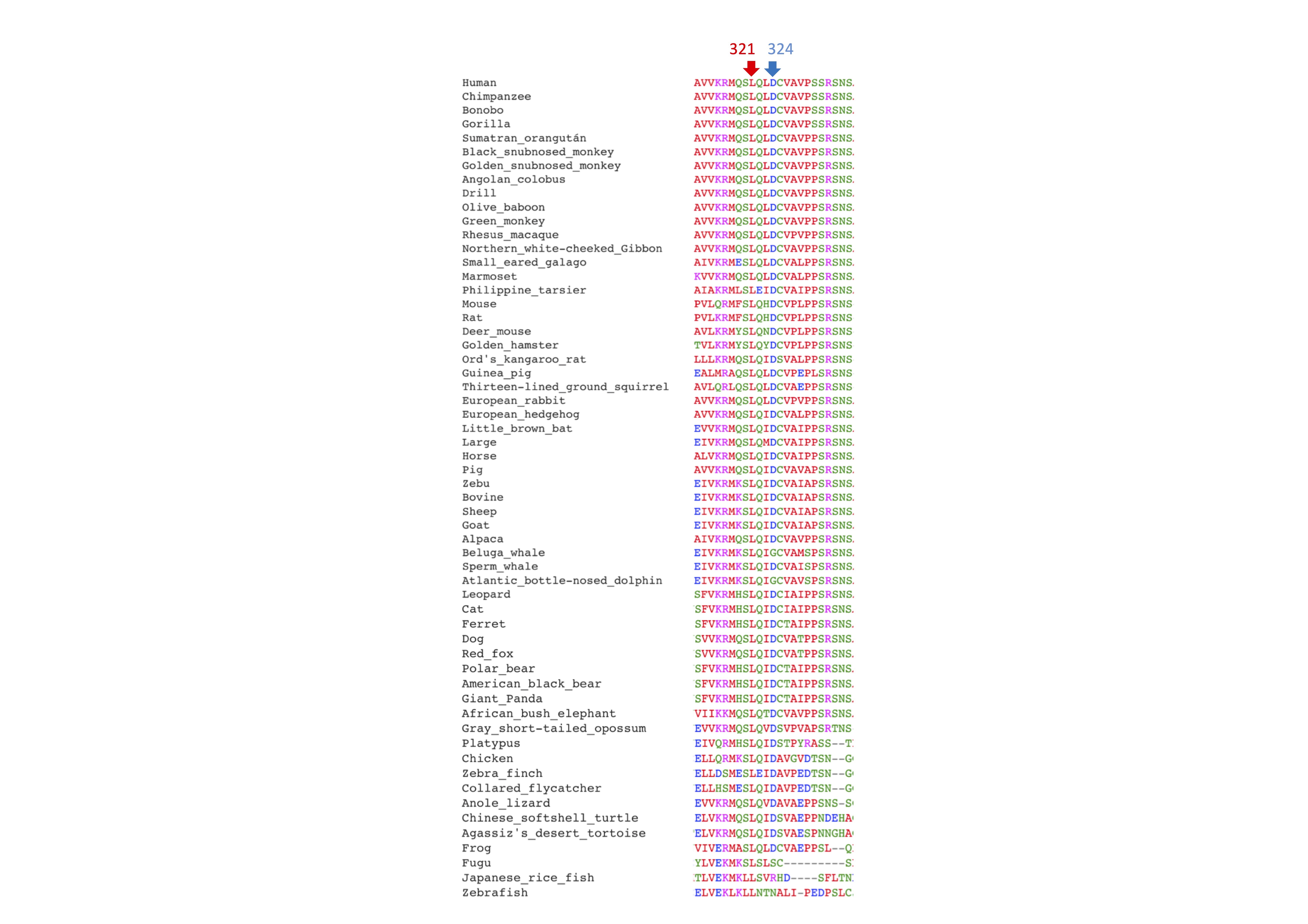

Supplement: Supplementary file 3 — (JPG 1836 kb) [file 10875_2022_1298_MOESM3_ESM.jpg]

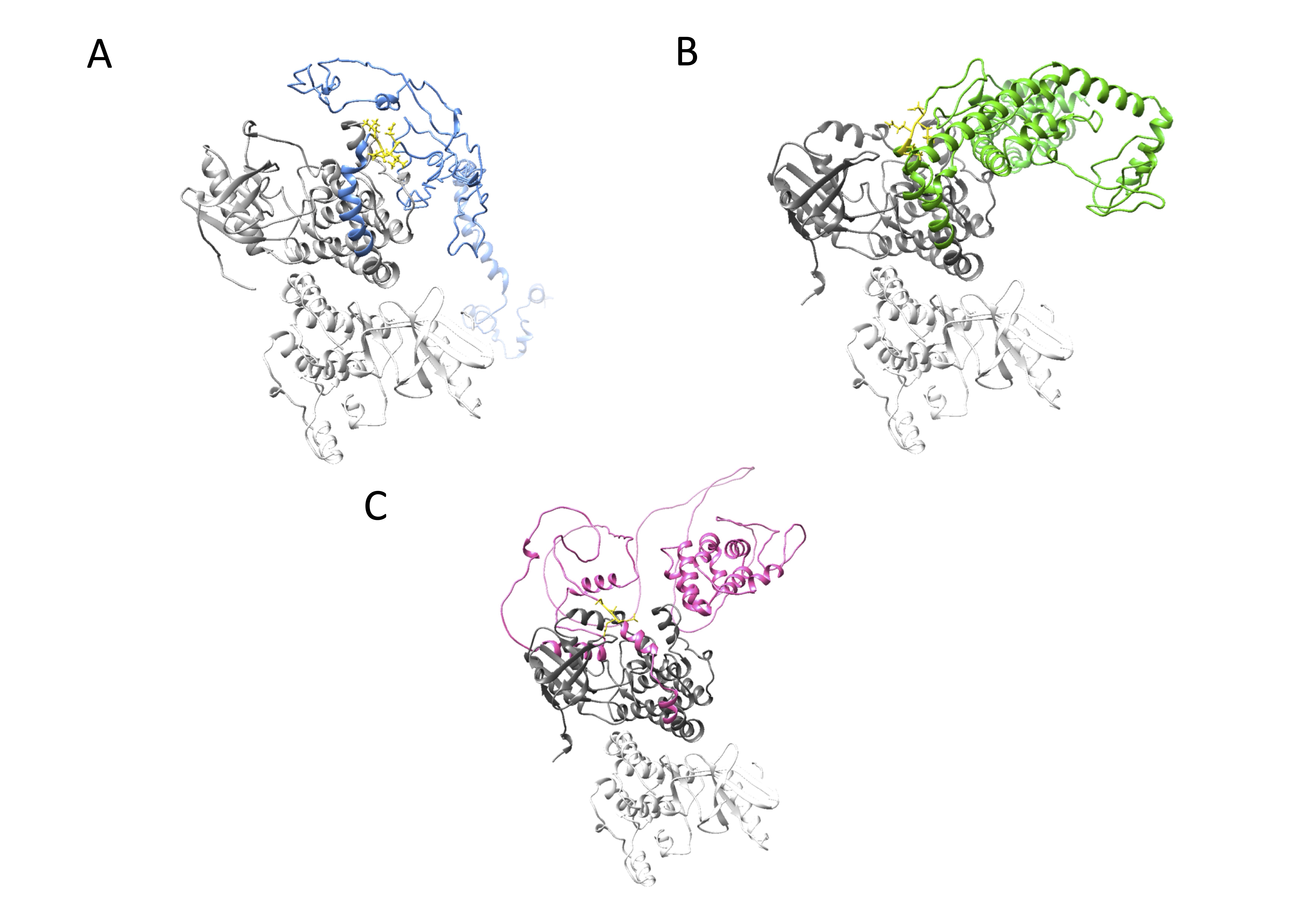

Supplement: Supplementary file 4 — (JPG 1536 kb) [file 10875_2022_1298_MOESM4_ESM.jpg]
